# Assessing post-operative dietary intake in older adult hip fracture patients: An observational study protocol

**DOI:** 10.1177/02601060241307768

**Published:** 2025-01-22

**Authors:** Helen Lloyd, Naomi Burn, Sarah Allison, Cecile Jones, John Derek Franklin

**Affiliations:** 1 School of Science, Technology and Health, 41872 York St John University, York, UK; 2 School of Health and Life Sciences, 5462 Teesside University, Middlesbrough, UK; 3 1067 University of South Australia, Adelaide, Australia; 4 Department of Sport, Exercise and Rehabilitation, 5995 Northumbria University, Newcastle Upon Tyne, UK

**Keywords:** Hip fracture, post-operative, older adult, dietary intake, food diary

## Abstract

**Background:** Fragility fractures of the hip are a common injury in England. Meeting post-operative resting energy expenditure (REE) needs are fundamental to recovery from trauma that with greater nutritional intake, post-operative complications and length of stay can be reduced. However, dietary intake can be overlooked when the goal is prompt surgery to reduce pain and lower the risks of mortality at 30 days and 1 year. **Aims:** The primary aim of this study is to observe if post-operative dietary energy intake (kJ/kcal) of older adult hip fracture patients meets their post-operative REE needs to mobilise post-surgery. Secondly, we aim to explore if there is a relationship between length of stay, comorbidity and post-operative complications in relation to dietary intake. **Methods and analysis:** Using a weighed food method, all food and fluid intake from the day of surgery until post-operative day three inclusive will be recorded for a cohort of 30 older adult hip fracture patients. Dietary intake per day will be compared against REE and macronutrient requirements. Baseline sociodemographic and medical history data will be obtained, along with admission data such as malnutrition screening and type of fracture. Regression analysis will be used to explore associations between dietary intake, post-operative complications and length of stay where indicated and to identify if there are areas for further dietary development in this specific patient group. **Ethics and dissemination:** The Health Research Authority approved this study (REC 24/NE/0034). Findings will be published in peer-reviewed, scientific journals and presented at academic conferences.

## Introduction

Older adult hip fracture patients are a significant surgical population in England, with over 65,000 cases registered in 2022 according to the National Hip Fracture Database (NHFD) (2023a) with average 30-day mortality rates at just over 6% (NHFD, 2023b). Approaches to in-patient care has seen marked developments in recent years, including orthogeriatric assessment, surgery within 36 hours of diagnosis and early mobilisation; strategies that have been incentivised via the Best Practice Tariff (National Health Service (NHS England, 2021) and hospital key performance indicators specified by the NHFD (2023c). However, an aging population and the long-term effects of COVID-19 has increased the risk of frailty (NHFD, 2023c), morbidity and polypharmacy, which can present challenges in providing even basic care needs, including supporting nutrition and dietary intake.

Malnutrition is a modifiable risk factor, and as outlined in an earlier review by Cochrane, malnutrition is prevalent in the hip fracture population (Avenell et al., 2016). However, the review explored supplementation strategies rather than dietary intake since patients ‘may have poor food intake while in hospital’. Conversely, studies of nutritional status have repeatedly demonstrated a correlation between malnutrition and lower functional outcome (Wong et al., 2021), mortality at 3, 6 and 12 months and the presence of post-operative delirium (Zanetti et al., 2019), however, focus has been on the most appropriate nutritional screening tool as opposed to opportunities for dietary improvement.

Current guidance in relation to specific nutritional support for hip fracture patients is limited, and rather the indication for nutritional support is based on a generic risk assessment for all adults regardless of condition or injury (NICE, 2024). The NHFD has measured nutritional risk assessment compliance since 2016 (NHFD, 2017), with the Malnutrition Universal Screening Tool (MUST) most used and is currently validated by both the British Association for Parenteral and Enteral Nutrition (BAPEN) and the National Institute for Health and Care Excellence (NICE) (BAPEN, 2003; NICE, 2024).

Research exploring the specific dietary intake of older adult hip fracture patients is also limited. Most recently, a short report of weighed lunch and dinner intake (excluding fluids), found dietary intake was poor (‘nutritionally vulnerable patients’ (*n* = 15) mean (SD): 643 (354) kcal/day), regardless of ‘good nutrition practice’ according to NHS England issued guidance (Sahota et al., 2023). However, methodological quality was poor owing to the type of study, with a lack of clarity regarding how intake was assessed, and the weighed element suggested to be only ‘total plate waste’ for the ward inclusive. Additionally, whilst the study claimed to focus on nutrition practice, there was no breakdown of macronutrients other than protein and no analysis of food diary data, but rather an exploration of food waste. In an earlier study that explored the dietary intake of hip fracture patients, only 59% of energy needs were met (week 2, 3-day intake; 4219 ± 319 kJ/day, kcal not stated), however, the authors stressed the importance of ‘early nutritional assessment’ but did not commence the food diary until post-operative week 2, and excluded those with cognitive impairment, which limits its generalisability (Nematy et al., 2006). Additionally, whilst the study analysed energy requirements according to basal metabolic rate (BMR), newer guidelines developed by the British Dietetic Association (BDA) and the Parenteral and Enteral Nutrition Specialist Group (PENG) state that resting energy expenditure (REE) is a more appropriate measure in estimating the nutritional requirements of adults when acutely unwell (Todorovic and Mafrici, 2018).

The standard approach in hospital for monitoring in-patient dietary intake is a visual estimation method (VEM), such as a ‘quarter’ or ‘half’ of a meal or expressed as a percentage (Heighington-Wansbrough and Gemming, 2022). However, the validity of this method is questionable on account of the potential for inaccuracy and variability between staff. In a systematic review of 14 studies using varying VEM methods, underestimation ranged from <2% to 53% in 5 studies, and overall, training was identified as a crucial factor in relation to compliance and accuracy (Hirsch et al., 2021). Although a weighed food diary was cited as the ‘gold standard’ for measuring dietary intake, in a ward context, this can be an arduous task.

Dietary intake in the immediate post-operative period is crucial in the care of surgical patients, on account of an altered metabolic state attributed to preoperative fasting, combined with an inflammatory response provoked by the trauma of injury (Hirsch et al., 2021). As opposed to minimally invasive surgery, a hip fracture repair requires a traditional ‘open’ approach that can exacerbate the surgical stress response, increase nutritional needs and prolong recovery. The preservation of muscle mass and meeting whole-body macronutrient energy needs is crucial following a hip fracture repair to allow for mobilisation. In a systematic review of 16 studies of hip fracture outcomes, early mobilisation resulted in a reduction in post-operative complications and length of hospital stay (LOS) (Aprisunadi et al., 2023). More specifically, protein and carbohydrates are crucial at each stage of perioperative care, of which carbohydrates are key to offset the effects of preoperative hypermetabolic catabolism as seen with preoperative fasting or in the presence of malnutrition, that subsequently, necessitates increased protein intake to offset muscle atrophy due to reduced mobility and surgical stress (Hirsch et al., 2021).

Overall, there is currently a lack of robust research concerning the specific post-operative dietary intake of older adult hip fracture patients. Whilst varying supplementation methods could be beneficial for those identified as malnourished, specific guidance regarding their nutritional management is limited. Therefore, it is warranted to observe post-operative dietary intake in the first instance, where there is potential to identify specific areas where recommendations for practice could be made.

### Research aims

The primary aim is to observe if the post-operative dietary energy intake (kJ/kcal) of older adult hip fracture patients meets their REE needs that would allow for basic movements, such as from bed to chair and initial mobilisation with a physiotherapist post-surgery.

Secondly, the aims of the study are to explore if there is a correlation between post-operative dietary intake and LOS; to explore the relationship between comorbidity (where reported) and post-operative dietary intake, according to dietary intake; and to explore (if) in the development of post-operative complications (including, delirium, infection, pressure ulcers and venous thromboembolism), there is a correlation with post-operative dietary intake.

### Hypothesis

1. We hypothesise that older adult hip fracture patients will not be meeting their daily REE needs.
2. We hypothesise that a reduced dietary intake due to participants being unable to meet their daily REE needs because of trauma, anaesthesia and surgery will have a negative impact on LOS and post-operative complications.
3. We hypothesise of an association between reduced dietary intake and a combination of nausea, delirium and pain.

To our knowledge, this is the first study to use a weighed food diary for older adult hip fracture patients from the day of surgery (day zero) to post-operative day three inclusive that will not require patient or ward staff recall. With data collection being conducted directly by the researcher, this will increase the accuracy of recording dietary intake.

## Methods

### Design

The design is a prospective study using a convenience sampling method. The novelty of this study is grounded in the approach of a post-operative weighed food diary. Rather than participant recall, ward staff recall or asking ward staff to weigh food (which was not feasible due to ward staff workload), the researcher will weigh and record the participants dietary intake which will therefore increase the accuracy of the data obtained.

Using this design and methodological approach may offset potential refusal to participate in the study as there is no foreseeable risks to the participant nor burden which could potentially lower attrition risks. The design of this study is aimed at exploring biopsychosocial factors associated with older adult hip fracture patients’ outcomes in relation to LOS and post-operative complications. In particular, the inclusion of participants that lack capacity and/or in the presence of comorbidity could provide crucial insight to a subgroup of an at-risk population that has been previously excluded. The study will be conducted in one phase of 4 weeks. Participants enrolled on the study will not be able to obtain their data after the study has been completed as they will be anonymised and assigned a unique reference number after LOS and discharge destination data has been obtained. With no second phase of the study, participants’ identifiable data will not be retained for future research purposes.

### Study sample

The study aims to recruit 30 older adult hip fracture patients admitted to an orthopaedic trauma ward for surgical repair of a hip fracture. A district general sized hospital was chosen as opposed to the scale of a trauma/transplant centre hospital to confer greater generalisability to the population in England with the findings from this study. A sample size of 30 was agreed by the research team based on the admissions data published by the NHFD for the specific study site and is reflective of a ‘typical’ average month of admissions to the hospital, according to 2021 and 2022 data. The decision to enrol 30 patients was primarily based on funding constraints, as there are currently no available funds to support a larger sample. This number was chosen to ensure we can gather detailed and meaningful data while adhering to our timeline and resources. The recruitment period will last for a continuous period of 4 weeks, and all eligible patients according to the inclusion and exclusion criteria will be invited into the study via the gatekeeper as they are admitted to the hospital ward. The standard diagnosis for a hip fracture is an x-ray on admission to the accident and emergency department, following which, the participant's care adheres to a specific ‘neck of femur (NOF) fracture pathway’ which is already in place at the study site.

### Inclusion criteria

The inclusion criteria for this study are adults aged 60 years and above that have sustained a hip fracture from a fall at standing height or less. There is no upper age limit, and no exclusions based on gender or ethnicity.

### Exclusion criteria

The exclusion criteria for this study are (a) pathological hip fractures, (b) traumatic hip fractures, for example sustained in a road traffic collision, (c) those that cannot speak English language due to obtaining informed consent and (d) those who are undergoing current oncologic treatment.

Due to the design of the study being observational, there are no further exclusions based on medical comorbidity, such as cardiovascular disease, diabetes and renal insufficiency. Those who present with cognitive decline will be approached for inclusion in the study via discussion between medical staff in consultation with a family member/friend whereby following an introduction of the study, their opinion will be sought as to whether they believe their family member or friend that has sustained a hip fracture would be likely to consent to taking part in the study if they had capacity.

### Screening

Each day, the researcher (HL) will check with the trauma coordinators on the ward responsible for the triage of hip fracture patients regarding new hip fracture admissions and discuss the potential for the participant to be approached for the study. On admission to the ward, the coordinators responsible for the admission and preparation of the hip fracture patient for surgery (the gatekeepers) will ask the participant if the researcher can be introduced to discuss the study.

### Enrolment procedure

With agreement, the researcher will discuss the study with the participant and family or carers if present and provide the participant with a patient information sheet (PIS) and consent form to read in full. After 1 hour, the researcher will return and obtain informed consent. The lead researcher for this study is a current registered healthcare professional with the Health and Care Professions Council in the UK and has training and experience of obtaining written informed consent. With the participants consent, the researcher, working with the specialist nurse will obtain data from the participant's medical records pertaining to outcome measures (Table 1).

**Table 1.** Outcome measures.

Variable	Instrument/scale
Age	Age will be measured in years based on the date of birth obtained from medical records.
Sex	As recorded in medical records.
Ethnicity	As recorded in medical records.
Original domicile	Defined as own home/care home/sheltered accommodation, according to medical records.
Mobility status prior to admission	Defined as independent/independent with 1 stick/independent with 2 sticks/independent with frame/wheelchair.
Nutritional screening	Screening assessment according to the Malnutrition Universal Screening Tool (0, 1 or 2).
BMI	Calculated by ward staff; where not possible, MUAC score.
REE	Calculated according to guidance (Todorovic and Mafrici, 2018)
Smoking status	Confirmed on admission; answered as yes or no.
Alcohol	Confirmed on admission; calculated as units per week.
Fracture type	Classified as intra-capsular or extra-capsular, confirmed on admission with an x-ray performed in the emergency department.
ASA Grade	Classified following anaesthetic pre-assessment by anaesthetist (1–5).
Acute length of stay	Measured from admission to discharge from the acute ward, in days.
Discharge location	Defined as original domicile/rehabilitation unit/care home.
Past medical history	Data will be obtained from medical records regarding cardiovascular, respiratory, diabetes, cognitive decline, arthritis and history of previous fractures.

### Food diary protocol

The weighed food diary will commence on the participants day of surgery (day zero) and will conclude at post-operative day three inclusive. The weighed food diary will consist of the researcher using an electronic digital kitchen scale with a weight graduation of 1 g (AccuWeight 201, maximum weight 5 kg) to weigh all items served to the participant before and after their mealtime. The researcher will not serve any food directly, with plates or bowls placed directly on the digital scale and recorded by the researcher. Should a food be packaged with a specified weight – for example a yoghurt, said weight on the packaging will be recorded. Fluid intake will be recorded according to each mealtime (e.g. a cup of tea or fruit juice) and the researcher will liaise with the ward staff to log how many jugs of water the participant has consumed daily, and if this has been accompanied with other items such as cordial. A baseline measurement of water jugs, cups and beakers will be taken prior to the study commencing.

The researcher will not be present nor disrupt the participant during their meal, and after their food tray has been retrieved, the researcher will weigh the plate if it is a complete meal (e.g. lasagne), or, if it is a multiple item meal (e.g. meat, potatoes and vegetables), the researcher will weigh the leftover components individually to ensure the validity of the weighed food method and to achieve the highest degree of accuracy possible. After mealtime has concluded, the researcher will briefly ask the participant if they have had any snacks between their meals. Any observed assistance with meals (such as a family member) will be confirmed with ward staff. Additionally, as part of the specific NOF pathway in place, all hip fracture patients regardless of MUST screening assessment are required to consume two oral nutritional supplements (Nutricia™ Fortisip Compact or Fortisip Compact Protein) daily. The researcher will liaise with the ward staff regarding consumption and additionally will record the amount consumed. Data will also be collected regarding the times of meals and the ratio of staff to patients on the ward during each mealtime.

The researcher will liaise daily with the participant's named nurse regarding complications that may develop post-operatively during the study period, such as delirium, pressure ulcers, infection and venous thromboembolism.

### Adverse events

Should a participant experience an adverse event during the study, such as a stroke, myocardial infarction, major haemorrhage requiring a blood transfusion, the participant will be removed from the study, which was agreed after consultation with the study site research department. If a participant is removed from the study due to an adverse event, any data collected up until that point will be kept in the study and anonymous and an adverse events record made. The participants’ end point in the study will be when LOS and discharge destination data is obtained by the researcher.

### Outcome measures

#### Baseline characteristics

Table 1 outlines the range of data that will be collected such as sociodemographic, nutritional status and medical history and will be analysed in Microsoft Excel.

#### Dietary intake

We will analyse if dietary intake is sufficient to meet estimated REE requirements and explore the effect of dietary intake as a predictor of LOS and post-operative complications. Current guidelines will be used to calculate REE, and we will use a factor of 23 kcal/kg actual body weight per day for ‘surgical fixation of a hip fracture’ according to the supporting evidence-based recommendations specified in the guidelines (Todorovic and Mafrici, 2018). Using macronutrient data for hospital meals provided by the trust, consumption and waste per meal will be calculated according to energy (kJ/kcal), protein, carbohydrates, fats and fibre. For any snacks consumed or foods not listed by the hospital, Nutritics v6.00 software will be used for macronutrient data (Nutritics, 2024).

#### Covariates

LOS data (measured in days) will be obtained from the participants medical records, and the occurrence of post-operative complications will be checked daily with the gatekeeper per participant.

The consent form for participants with capacity and the Consultee Form for participants without capacity were approved by the NHS Ethics Committee. A registered dietitian that is not linked to the study site (CJ) reviewed the study protocol, weighed food diary chart and outcome measures.

### Data storage and analysis

Participant identifiable data (name, date of birth and hospital number) will be used to create a unique identifier that will ensure the correct food diary data is recorded against the correct patient, when accounting for a participant's potential movement between rooms during their ward stay. Once the participant's LOS and discharge destination data has been obtained, the participant's data will be anonymised and assigned a unique reference number. All participant data will be recorded and held within the trust site to maintain confidentiality and the transfer of patient information once assigned an identifier will be from the research department to the researcher's secure email.

Baseline nutritional requirements for REE, total energy expenditure (TEE), protein, carbohydrates and fat nutritional requirements per day will be calculated according to the specific guideline formula (Todorovic and Mafrici, 2018). In the instance where BMI is outside of the range 18.5 to 30 kg/m^2^, calculation adjustments will be made as per the guideline recommendations (Todorovic and Mafrici, 2018). Regarding physical activity and diet-induced thermogenesis (PAL), a combined factor range of 1.0 to 1.10 will be used, which the guidelines state is a ‘post-surgery factor’ (Todorovic and Mafrici, 2018). In cases where there is a range specified, calculations of nutritional requirements will be made at both the lower and higher scale. Demographic variables will be presented in descriptive summaries such as mean ± SD and median for continuous variables and percentages for categorical variables. For statistical analysis we will present descriptive statistics for REE met per day and to make inferences, we will take an average of the proportion of REE intake of the total food diary.

Multiple regression analysis will be used to explore if REE is a predictor of LOS, with post-operative complications and medical history used as confounders along with those who have capacity or not. Due to a small sample size, we acknowledge that caution should be taken with secondary subgroup analysis. In addition, where REE is met or exceeded, participants food diaries will be scrutinised to explore if this is attributed to a specific category of food (e.g. a high-fat dessert item) and where REE is not met, food diaries will be scrutinised to explore if this is related to complications reported.

### Ethics and dissemination

Ethical approval and permissions for this study were sought from:

- NHS Ethics – approval was granted by the Health Research Authority (REC reference: 24/NE/0034)
- This study is registered at ClinicalTrials.gov (NCT06451679)

The study will be conducted in accordance with the principles of the Health Research Authority (HRA) Good Clinical Practice (NHS HRA, 2020) and adhere to appropriate legislation regarding the protection of data for participants in the study (NHS HRA, 2021).

Written informed consent will be obtained from participants that have been assessed by a medical doctor as having capacity on admission following a discussion of the study between the participant and the researcher. Should a participant lack capacity, the researcher will approach the participant for inclusion in the study after a discussion with medical staff (the Consultee) regarding suitability. Ward medical staff will be given a briefing before the study commences and issued with a Consultee Information Sheet (CIS). If the participant is suitable, the Consultee will introduce the researcher to a family member/friend whereby with an introduction of the study, their opinion will be sought as to whether they believe their family member or friend that has sustained a hip fracture would be likely to consent to taking part in the study if they had capacity. If they agree, the Consultee will complete a specific ‘Consultee Form for Participants Lacking Capacity’. All participants will be informed that they can withdraw from the study at any time with no reason required, and, should a participant lack capacity initially and subsequently regain capacity, they will be informed immediately of the study and will be able to remain in the study, withdraw from study and withdraw their data, or withdraw from the study and allow the researcher to keep their data up until that point.

Results from the observation study will be reported using the Strengthening the Reporting of Observational Studies in Epidemiology (STROBE) guidelines (Von Elm et al., 2007) and disseminated at relevant scientific conferences and by publication of peer-reviewed manuscripts.

## Discussion

To our knowledge, this is the first observational study of older adult hip fracture patients using a weighed food diary for all dietary intake from day zero until post-operative day three inclusive. The protocol will add to the literature to date by identifying if older adult hip fracture patients are able to meet their REE baseline needs following surgical repair of a hip fracture and secondly, if there is a correlation between LOS, comorbidity and post-operative complications in relation to dietary intake.

Previous research to date has either focussed on the effects of supplementation in relation to dietary intake (Avenell et al., 2016) or the sensitivity of malnutrition screening tools (Zanetti et al., 2019). Whilst further research regarding dietary intake is necessitated at every stage of hip fracture care to inform the update of clinical guidance, it is hoped that the results of this study will support the education and training of those involved directly in the dietary care of older adult hip fracture patients.

### Strengths and limitations of this study

The data from the study could indicate future areas for research in older adult hip fracture patients, for instance, at which stage of the perioperative pathway should more nutritional support be targeted. Whilst a weighed food diary may not be feasible long-term in daily ward care on accounts of its laborious nature, the higher degree of accuracy will highlight areas of care for further improvement and research focus. Given the limited sample size, we acknowledge that caution should be taken when generalising the findings to the population. Additionally, as this is a single-phase study without follow-up to assess long-term outcomes, this limits the evaluation of post-operative dietary intake on rehabilitation. Without an evaluation of post-operative dietary intake and its effectiveness in meeting the nutritional needs of older adult hip fracture patients, the risk of a generalised approach to the dietary intake of older adult hip fracture patients could lead to a greater burden on the NHS.
